# Supplementary material for: Association between preoperative levels of 25-hydroxyvitamin D and hospital-acquired infections after hepatobiliary surgery: A prospective study in a third-level hospital
Source: PLoS One. 2020 Mar 26;15(3):e0230336. doi: 10.1371/journal.pone.0230336 (PMC7098583; doi:10.1371/journal.pone.0230336)
Supplement: Attachment — Submitted filename: Response to reviewers.docx [file pone.0230336.s001.docx]

Dear Editor ,

We thank the editor and the reviewers for their corrections and comments, which we feel have helped us to significantly improve the manuscript. We hope that you find the revised version of the manuscript suitable for publication in PLOS One.

Below is a point-by-point list indicating the changes made to the manuscript based on your comments and those of the reviewers.

Editor comments:

1. **Abstract- They have noted Discussion at the end, which needs to be replaced with “Conclusions” of the study.**

The heading of this paragraph has been changed “Conclusions”.

2. **(p.6; lines 133-139) delete the ‘title’ of the manuscript here.**

The title on page 6 has been deleted as suggested.

3. **Also, given the previous studies in the field, the information provided “Introduction” needs to be detailed with more citations.**

More citations have been added to the Introduction section in order to provide a better overview of previous studies in the field .

4. **Methods- clarify the statement that the study with patient population is approved with institutional IRB.**

CEICA (the Research Ethics Committee of Aragon, Spain) approved our study and revised our informed consent form before starting. This review board is responsible for revising the ethical standards and financial disclosures of every study performed in Aragon. Additional information has been added to the revised manuscript to clarify this issue.

5. **Also, note separately a section of the “Statistical analysis” of the results. Also, note a separate paragraph on ‘vitamin D’ assays used to consider deficiency as per the American Endocrine Society guidelines (ex., citation #7) in Methods.**

The methods section has been reorganized in line with these suggestions. A new statistical analysis section has been added, as well as an additional paragraph describing the vitamin D assays and providing more information about the definition of optimal vitamin D levels.

6. **Results- please provide a clear rationale of the study undertaken given other workers findings on the subject in the field**.

We explained that we did not find a significant association with cardiovascular disease because of sample size, sample population and study design.

7. **p.18; line 353-Remove citation name “Youssef et al” as it belongs only to citation #20, but it is noted as citations 6,17,20.**

We thank the editor for noting this citation error, which been corrected.

8. **Figure.1&2 embedded in the text are fine but seems repeated providing eps version again at the end after the References. Provide more details in the legends.**

In the revised manuscript more information has been added to the figure legends for greater ease of interpretation.

Journal requirements:

1. **Please ensure that your manuscript meets PLOS ONE's style requirements, including those for file naming. The PLOS ONE style templates can be found** [**http://www.plosone.org/attachments/PLOSOne_formatting_sample_main_body.pdf**](http://www.plosone.org/attachments/PLOSOne_formatting_sample_main_body.pdf) **and** [**http://www.plosone.org/attachments/PLOSOne_formatting_sample_title_authors_affiliations.pdf**](http://www.plosone.org/attachments/PLOSOne_formatting_sample_title_authors_affiliations.pdf)

The file name and paragraph headings have been changed in accordance with style requirements.

2. **Please provide additional details regarding participant consent. Since the requirement for written informed consent was waived by the ethics committee, please ensure that you have discussed whether all data collected in this study were fully anonymized before you accessed them.**

Additional information has been added describing informed consent and data collection/anonymization.

3. **In your Methods section, please provide additional information about the participant recruitment method and the demographic details of your participants. Please ensure you have provided sufficient details to replicate the analyses such as: a) the recruitment date range (month and year), b) a description of any inclusion/exclusion criteria that were applied to participant recruitment, c) a description of how participants were recruited, and d) descriptions of where participants were recruited and where the research took place.**

Additional information on the inclusion and exclusion criteria, recruitment date range, and patient demographics has been added.

4. **At this time, we ask that you please provide additional information in your Methods section about the methodology used to conduct the chemiluminescent microparticle immunoassay (CMIA) to measure vitamin D concentrations in the patients serum samples**.

Additional information has been added describing the CMIA procedure.

5. **Thank you for stating the following financial disclosure: "NO". Please provide an amended Funding Statement that declares *all* the funding or sources of support received during this specific study (whether external or internal to your organization) as detailed online in our guide for authors at** [**http://journals.plos.org/plosone/s/submit-now**](http://journals.plos.org/plosone/s/submit-now)**.  Please state what role the funders took in the study.  If any authors received a salary from any of your funders, please state which authors and which funder. If the funders had no role, please state: "The funders had no role in study design, data collection and analysis, decision to publish, or preparation of the manuscript." Please include your amended statements within your cover letter; we will change the online submission form on your behalf.**

None of the authors received any external funding or additional financial assistance from their respective institutions. This information has been added to the manuscript and the cover letter.

6. **Thank you for stating the following in your Competing Interests section: "NO" Please complete your Competing Interests on the online submission form to state any Competing Interests. If you have no competing interests, please state "The authors have declared that no competing interests exist.", as detailed online in our guide for authors at** [**http://journals.plos.org/plosone/s/submit-now**](http://journals.plos.org/plosone/s/submit-now) **This information should be included in your cover letter; we will change the online submission form on your behalf.**

The authors have declared that no competing interests exist.

7. **Please amend your list of authors on the manuscript to ensure that each author is linked to an affiliation. Authors’ affiliations should reflect the institution where the work was done (if authors moved subsequently, you can also list the new affiliation stating “current affiliation:….” as necessary).**

The list of authors has been amended accordingly to provide affiliations in each case.

8. **Your ethics statement must appear in the Methods section of your manuscript. If your ethics statement is written in any section besides the Methods, please move it to the Methods section and delete it from any other section. Please also ensure that your ethics statement is included in your manuscript, as the ethics section of your online submission will not be published alongside your manuscript.**

The ethics statement has been moved to the methods section.

Reviewer comments:

1. **In line 161 authors refer previous studies similar to the submitted manuscript. It would be good to have a short explanation stating how this work in different to those.**

A short explanation describing how this study differs to those previously published has been added to the revised manuscript.

2. **In the legend of the first table the meaning of the abbreviations (ASA, COPD) is not included.**

The legend of Table 1 has been edited to include the meanings of each of the abbreviations used.

3. **Better explanation of how the percentiles in table 1 were calculated is needed.**

Further information has been added to describe the process of calculating the percentiles.

4. **In line 235 authors mentioned Score II, but never mentioned this score in the manuscript. A brief explanation of what this means and why it is relevant to this work will be helpful to follow the manuscript.**

Score II refers to the ASA index score. This information is now included in the methods section.

5. **In the third paragraph of the discussion is mentioned that other studies suggest a link between vit-D levels and heart disease. But this study does not observe statistical significance here. A possible reason for the different results should be included. Also the 4th paragraph of the discussion refers to 3rd paragraph (line 365) so the new paragraph is not really needed.**

We have corrected the third and the fourth paragraphs of the discussion. We were unable to detect an association between cardiovascular disease and vitamin D levels due to the sample size, sample population, and study design.

Estefania Laviano Martinez
